# *Arabidopsis* MKS1 Is Involved in Basal Immunity and Requires an Intact N-terminal Domain for Proper Function

**DOI:** 10.1371/journal.pone.0014364

**Published:** 2010-12-28

**Authors:** Klaus Petersen, Jin-Long Qiu, Juri Lütje, Berthe Katrine Fiil, Sidsel Hansen, John Mundy, Morten Petersen

**Affiliations:** Department of Biology, University of Copenhagen, Copenhagen, Denmark; Umeå Plant Science Centre, Sweden

## Abstract

**Background:**

Innate immune signaling pathways in animals and plants are regulated by mitogen-activated protein kinase (MAPK) cascades. MAP kinase 4 (MPK4) functions downstream of innate immune receptors via a nuclear substrate MKS1 to regulate the activity of the WRKY33 transcription factor, which in turn controls the production of anti-microbial phytoalexins.

**Methodology/Principal Findings:**

We investigate the role of MKS1 in basal resistance and the importance of its N- and C-terminal domains for MKS1 function. We used the information that *mks1* loss-of-function partially suppresses the *mpk4* loss-of-function phenotype, and that transgenic expression of functional MKS1 in *mpk4*/*mks1* double mutants reverted the *mpk4* dwarf phenotype. Transformation of *mks1*/*mpk4* with mutant versions of MKS1 constructs showed that a single amino acid substitution in a putative MAP kinase docking domain, MKS1-L32A, or a truncated MKS1 version unable to interact with WRKY33, were deficient in reverting the double mutant to the *mpk4* phenotype. These results demonstrate functional requirement in MKS1 for the interaction with MPK4 and WRKY33. In addition, nuclear localization of MKS1 was shown to depend on an intact N-terminal domain. Furthermore, loss-of-function *mks1* mutants exhibited increased susceptibility to strains of *Pseudomonas syringae* and *Hyaloperonospora arabidopsidis*, indicating that MKS1 plays a role in basal defense responses.

**Conclusions:**

Taken together, our results indicate that MKS1 function and subcellular location requires an intact N-terminus important for both MPK4 and WRKY33 interactions.

## Introduction

Plants are constantly exposed to a broad range of microbes and have evolved an innate immune system to defend against invading pathogens [1], [2]. Part of this system is based on the perception of pathogen- or microbe-associated molecular patterns (PAMPs or MAMPs) through pattern recognition receptors (PRRs) at the cell surface. A common mechanism to transduce such signals into cellular responses is the activation of mitogen-activated protein kinase (MAPK) cascades. These cascades consist of MAPKKK-MAPKK-MAPK modules that link upstream receptors and downstream targets leading to rapid activation of defense responses upon recognition of invading pathogens [3], [4].

Loss of *Arabidopsis* MAP kinase 4 (MPK4) activity leads to dramatic changes in gene-expression, elevated levels of the phytohormone salicylic-acid (SA), increased resistance to biotrophic pathogens, and *mpk4* loss-of-function mutants are extreme dwarfs [5]. The resistance-associated phenotypes of *mpk4* are suppressed by mutations in two defense regulators, ENHANCED DISEASE SUSCEPTIBILITY 1 (EDS1) and PHYTOALEXIN DEFICIENT 4 (PAD4) [6]. In contrast, MPK4 seems to be required for the expression of defense genes that rely on the phytohormones ethylene and jasmonate (ET/JA) and efficient resistance against necrotrophic pathogens. Interestingly, EDS1 was found to be responsible for blocking *PDF1.2* expression in *mpk4* single mutants, because *mpk4*/*eds1* double homozygotes express *PDF1.2* in response to jasmonate [6].

MAP kinase substrate 1 (MKS1) was previously identified as a MPK4 substrate, and analysis of transgenic plants, and transcript profiling indicated that MKS1 was required for full resistance in *mpk4* mutants [7]. MKS1 was also found to interact *in vivo* with the transcription factor WRKY33, and may function as an adaptor linking MPK4 activity to WRKY-regulated gene expression [8]. WRKY transcription factors constitute a family of defense-related factors that bind W-box sequences in the promoters of pathogen-induced genes, including WRKY genes themselves [9], [10]. It was shown that MPK4 and MKS1 associate in a complex with WRKY33, and that activation of MPK4 and phosphorylation of MKS1 upon infection leads to release of MKS1 and WRKY33 from MPK4. Consequently, WRKY33 targeted the promoter of *PHYTOALEXIN-DEFICIENT3* (*PAD3*), encoding a cytochrome P450 monooxygenase required for the production of the antimicrobial phytoalexin camalexin [8].

MKS1 contains a plant-specific VQ motif that is found in about 35 predicted *Arabidopsis* proteins [11]. These proteins are generally small and share little significant similarity to other sequenced or predicted proteins. Other than the regions containing the conserved VQ motif, their primary structures also appear to be highly diverse [11].

MAP kinases phosphorylate their substrates on conserved Ser/Thr-Pro phosphoacceptor sites [12]. However, targeting of a MAP kinase to a specific substrate does not only depend on the phosphoacceptor site, but is also mediated by physical interaction between the kinase and MAP kinase docking domains present on substrate protein [13]. Docking domains have been identified in numerous MAP kinase substrates, and consist of a K/R_1-4_-X_1-6_-ϕ_A_-X-ϕ_B_ module, where ϕ_A_ and ϕ_B_ are hydrophobic residues (leucine, isoleucine or valine) [13].

MKS1 contains 12 Ser–Pro and our initial analyses indicated that MPK4 phosphorylated MKS1 *in vitro* on Ser30 and/or Ser72 and perhaps on other Ser–Pro sites throughout MKS1 [7]. In a more recent paper we used mass spectrometry to identify *in planta* activated MPK4 phosphorylation of MKS1. However, using this technique we did not detect phosphorylation of Ser30, but only phosphorylation of Ser72, Ser108, and Ser120 [14].

Here we show that MKS1 plays a pivotal role in basal defense responses in *Arabidopsis* as loss-of-function *mks1* mutants exhibit increased susceptibility to strains of *Pseudomonas syringae* and *Hyaloperonospora arabidopsidis*. In addition, we show that MKS1 function and subcellular location requires an intact N-terminus important for both MPK4 and WRKY33 interactions.

## Results

### *mks1* suppresses the *mpk4* dwarfism phenotype

**Figure 1:**
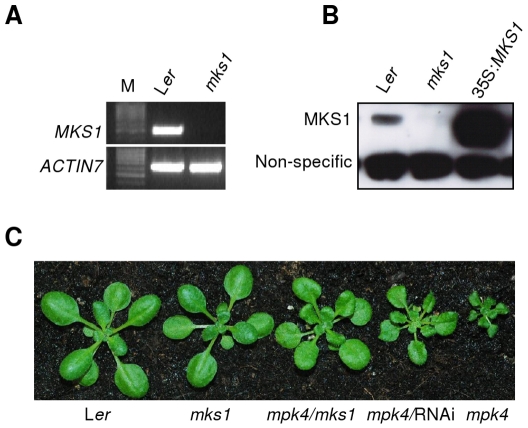
Characterization of *MKS1* Ds-insertion line (GT.108403). (**A**) RT-PCR analysis of *MKS1* transcript in L*er* and *mks1* mutant. *ACTIN7* is used as control. Lane M is a 100 bp marker. (**B**) Western blot analysis of MKS1 protein in *mks1* mutant. 35S:*MKS1* is used as positive control. Non-specific bands serve as loading control. (**C**) *mks1* can partially rescue the dwarf phenotype of *mpk4.* Three week-old plants with the indicated genotypes were grown under short-day conditions.

We previously demonstrated that reduced accumulation of *MKS1* mRNA via RNAi (*MKS1*-RNAi) partially suppressed the dwarfism and disease resistance conferred by *mpk4* mutation in homozygous *mpk4* expressing *MKS1*-RNAi [7]. However, the resistance phenotype of *MKS1*-RNAi was not significantly different from wild-type [7]. This may have been because MKS1 does not contribute to basal defense, or that the levels of *MKS1* mRNA in *MKS1*-RNAi plants were sufficient to mask such a phenotype. To distinguish between these two possibilities, we obtained an *MKS1* Ds-insertion line (GT.108403). RT-PCR identified *MKS1* transcripts in the parental wild-type background L*er* but not in the *mks1* insertion mutants (Figure 1A). Furthermore, immuno-blotting with an MKS1-specific antibody [7] demonstrated the presence of MKS1 protein in L*er* and in transgenic plants in which *MKS1* expression is controlled by the *CaMV 35S* promoter, but failed to detect MKS1 protein in the *mks1* insertion line (Figure 1B). The *mks1* null mutant was expected to suppress the *mpk4* phenotype at least to the same level as seen with *MKS1*-RNAi [7]. Therefore, the *mks1* insertion allele was crossed into the *mpk4* background and double mutant *mpk4*/*mks1* progeny confirmed by PCR (data not shown). Notably, the suppression of *mpk4* dwarfism and leaf epinasty by the *mks1* insertion allele was stronger than what we originally found with *MKS1*-RNAi (Figure 1C).

### *mks1* mutants exhibit decreased basal resistance to pathogens

**Figure 2:**
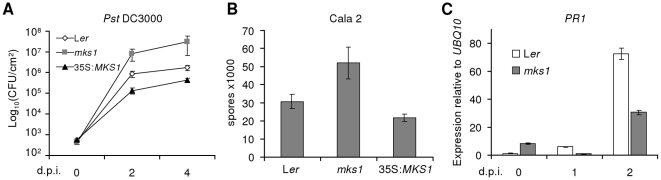
The *mks1* mutant is hypersusceptible to *P. syringae* and *H. arabidopsidis*. (**A**) Susceptibility of L*er*, *mks1* and 35S:*MKS1* plants to *Pst* DC3000. (**B**) Number of asexual spores 6 days after inoculation by spray of virulent *H. arabidopsidis* Cala 2 isolate (10^5^ spores/mL). Similar results were obtained in independent experiments. (**C**) *PR1* mRNA levels measured by real-time PCR in L*er* and *mks1* after *Pst* DC3000 infection. Means ± SD are shown. dpi: days post infection.

Since the *mks1* mutant is a stronger suppressor of *mpk4* than *MKS1*-RNAi, MKS1 could be engaged in cellular processes, including general plant defense responses, not detected via the RNAi approach. We therefore monitored the disease susceptibility of *mks1* infected with *P. syringae Pst* DC3000. Time-course triplicate assays revealed that, 2 and 4 days after inoculation with virulent *Pst* DC3000, bacterial growth in leaves of soil grown *mks1* mutant plants was up to 10-fold higher compared to L*er* wild-type (Figure 2A). To verify this finding, we extended the analysis with another pathogen class by inoculating *mks1* mutants with a virulent isolate (Cala 2) of the oomycete *Hyaloperonospora arabidopsidis*. Colonization by the virulent Cala 2 isolate was considerably more pronounced in *mks1* compared to L*er*, as revealed by the number of spores per plant (Figure 2B) and by macroscopic disease symptoms (data not shown). This suggests that loss of *MKS1* affects induced defense responses. We therefore examined the accumulation of the pathogenesis-related marker *PR1* mRNA by real-time RT-PCR in *mks1* mutants after challenge with virulent *P. syringae*. However, *PR1* accumulated to much lower levels in *mks1* compared to wild-type L*er* leaves 2 days after infection (Figure 2C). These results indicate that MKS1 plays a role in maintaining basal resistance.

### Overexpression of MKS1 leads to susceptibility to the necrotrophic pathogen *B. cinerea*

**Figure 3:**
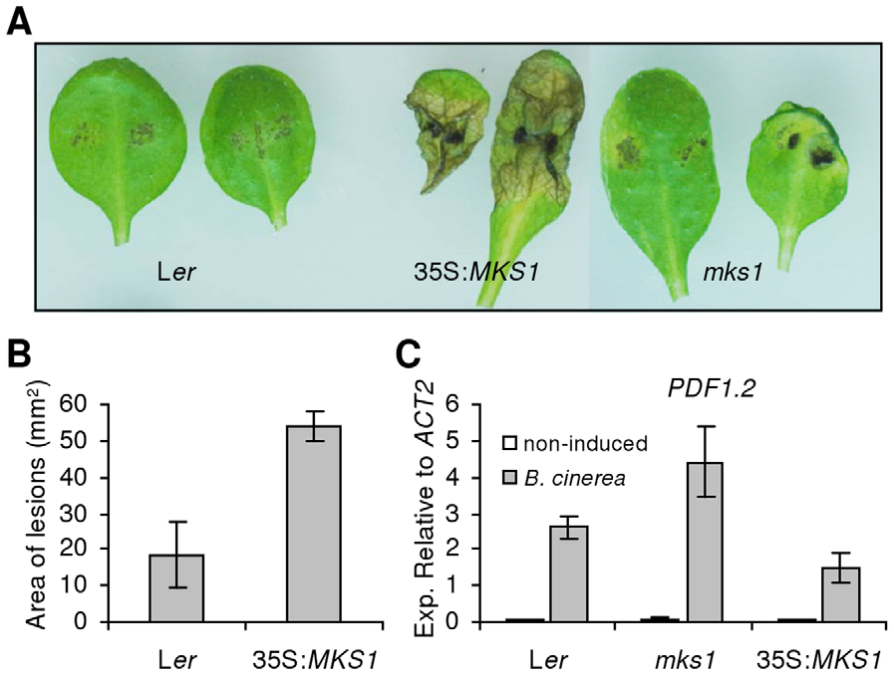
Overexpression of *MKS1* is susceptible to *B. cinerea*. (**A**) Disease symptoms showing fungal growth after inoculation with 2.5×10^5^ *B. cinerea* spores/mL. (**B**) Area of spreading lesions was determined after 60 h. Data represent average area of >10 lesions ± standard error. (**C**) Real-time PCR detection of *PDF1.2* mRNA after inoculation with *B. cinerea*. Samples were tested in triplicate and normalized to *ACTIN2*. Means ± SD are shown.

To further characterize the role of MKS1 in disease resistance, we included a necrotrophic pathogen in our analysis and inoculated both *mks1* mutants and transgenic plants overexpressing *MKS1* (35S:*MKS1*) with the fungus *B. cinerea*. The 35S:*MKS1* plants displayed severe tissue damage 5 days after inoculation (DAI) compared to both *mks1* and the L*er* wild-type (Figure 3A). The size of disease lesions on inoculated leaves of 35S:*MKS1* was increased compared to L*er* wild-type (Figure 3B) and *mks1* (data not shown). Moreover, fewer spreading lesions appeared in *mks1* plants than in 35S:*MKS1* or L*er* wild-type after inoculation (data not shown). To determine whether enhanced susceptibility to *B. cinerea* in 35S:*MKS1* is associated with altered defense responses against necrotrophs, we determined the expression of the defense marker gene *PDF1.2* following *B. cinerea* inoculation. The expression of *PDF1.2* was significantly reduced in 35S:*MKS1* compared with wild-type, whereas *PDF1.2* was highly elevated in *mks1* (Figure 3C). Collectively, these data indicate that MKS1 negatively regulate resistance responses required to combat the necrotrophic pathogen *B. cinerea*.

### MPK4 and WRKY33 interact with different regions of MKS1

**Figure 4:**
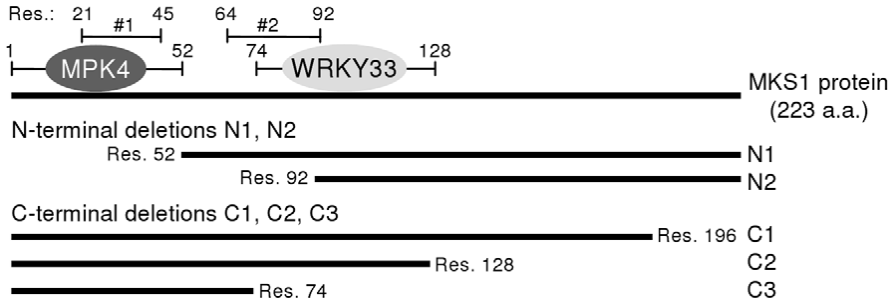
Yeast two-hybrid analysis for MKS1, MPK4 and WRKY33 proteins. Schematic representation of MKS1 truncations used in this study. MKS1: full length MKS1, N1: MKS1ΔN1, N2: MKS1ΔN2, C1: MKS1ΔC1, C2: MKS1ΔC2, C3: MKS1ΔC3. Summary of yeast two-hybrid analysis, MPK4 interacts with domain 1 (#1) of MKS1, WRKY33 interacts with domain 2 (#2) of MKS1.

We previously showed that MKS1 interacts *in vivo* with MPK4 and WRKY33 [7], [8]. To further specify which domains of MKS1 are important for these interactions, we performed a directed yeast two-hybrid analysis with two N-terminal deletions and three C-terminal deletions (Figure 4). N-terminal deletion 1, MKS1ΔN1, lacks domain 1 containing a putative MAP kinase docking domain [13]. MKS1ΔN2, lacks both domain 1 and domain 2 (containing a conserved VQ motif) [7]. MKS1ΔC3, contains only domain 1 and part of domain 2. MKS1ΔC2 consist of both domains, while MKS1ΔC1 only lacks the 27 most C-terminal amino acids of MKS1. Table 1 summarizes the results of the yeast two-hybrid screen. Unfortunately, the MKS1ΔN2 truncation was self-activating and was therefore omitted from further analysis. Nevertheless, we found that MPK4 interacted with all C-terminal truncations, but not with MKS1ΔN1, indicating that domain 1 of MKS1, containing the putative MAP kinase docking domain, is necessary for interaction of MPK4 with MKS1 (Figure 4). WRKY33 interacted with MKS1ΔN1, MKS1ΔC1 and MKS1ΔC2, but not with the shortest version of MKS1, MKS1ΔC3, suggesting that WRKY33 interacts with the region of 74 to 128 residues of MKS1 that includes domain 2 with the VQ motif (Figure 4).

**Table 1:** Yeast two-hybrid analysis for MKS1, MPK4, WRKY25 and WRKY33 proteins.

pBD/pAD					
vector	MKS1ΔN1	MKS1ΔN2	MKS1ΔC1	MKS1ΔC2	MKS1ΔC3
MPK4	-	N/A	+	+	+
WRKY25	+	N/A	+	-	-
WRKY33	+	N/A	+	+	-

Yeast was spotted onto SD-2 medium lacking trp and leu and SD-4 medium also lacking ade and his, and all combinations that grew on both media were scored as an interaction (+).

No interaction is indicated by (-). MKS1ΔN2 was selfactivated.

N/A not applicable.

### MPK4 only interacts with an intact N-terminal domain of MKS1

**Figure 5:**
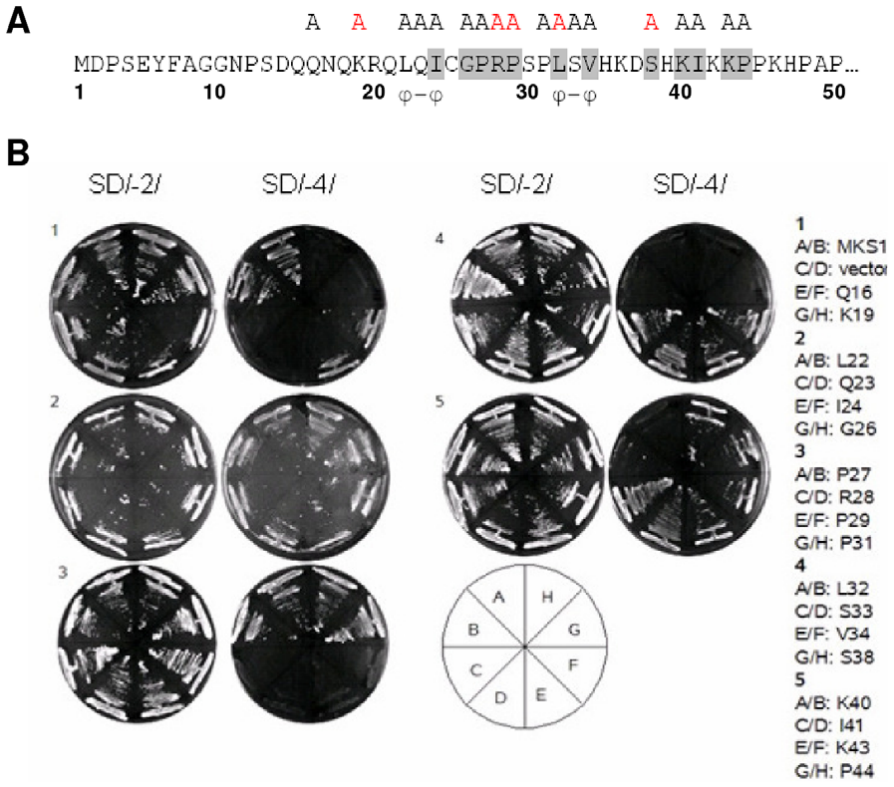
Yeast two-hybrid analysis for MKS1 point mutants and MPK4. (**A**) Alanine substitutions in the N-terminus of MKS1 are marked with an **A** above them. Black **A** indicates that the substitution did not interfere with the MKS1-MPK4 interaction in a yeast two-hybrid analysis. Red **A** indicates that the substitution resulted in loss of interaction. Residues of MKS1 that are conserved in other proteins (see Figure S1) are marked with a grey background. Two putative hydrophobic motifs (docking motifs) are marked below as ϕ-ϕ. (**B**) Yeast was spotted onto SD-2 medium lacking trp and leu and SD-4 medium also lacking ade and his, and all combinations that grew on both selection media were scored as an interaction (+). No interaction is indicated by (–).

To investigate the importance of the protein sequence in the putative MAP kinase docking domain of MKS1, we made 18 alanine substitutions (Figure 5A) and conducted a directed yeast two-hybrid assay with MPK4 as bait. The amino acids to be substituted were selected by their level of conservation among different plant MKS1 homologs (Figure S1). Of the 18 alanine substitutions, six were found to be important for interaction (Figure 5B). Two possible ϕ_A_-X-ϕ_B_ motifs exist in the N-terminus of MKS1: Leu22-Gln23-Ile24 and Leu32-Ser33-Val34 (Figure 5A). The ϕ_A_-X-ϕ_B_ motif inserts into the docking groove on the MAP kinase, and alanine substitutions in this motif have been shown to block activation of the MEF2A transcription factor by the MAPK p38 in mammalian cells [15]. Since substituting Leu32 with alanine caused disruption of binding and no such effect is observed for substitutions of Leu22, Gln23 or Ile24, it is likely that Leu32-Ser33-Val34 constitutes the relevant ϕ_A_-X-ϕ_B_ motif. This is also supported by the observation that Leu32 and Val34 are highly conserved among the different MKS1 homologs, whereas Leu22 shows no conservation at all (Figure S1). This points to the existence of a docking domain at the N-terminus of MKS1 fitting the K/R_1-4_-X_1-6_-ϕ_A_-X-ϕ_B_ module [16]. However, we previously showed that MPK4 phosphorylates MKS1 *in vitro* on Ser30 [7], and mammalian studies have shown that the classical docking motif binds to a region that lies on the other side of the protein to a phosphorylation site [17]. These results would exclude a classical docking domain within a phosphorylation site. However, a recent study shows that *in planta* activation of MPK4 by inoculation with *P. syringae* DC3000 expressing the AvrRpm1 avirulence factor only phosphorylates MKS1 on Ser72, Ser108, and Ser120, and not on Ser30 [14]. If this is the case, it is probable the N-terminus of MKS1 contains a ‘classical’ docking domain.

### An intact N-terminus is required for MKS1 nuclear localization

**Figure 6:**
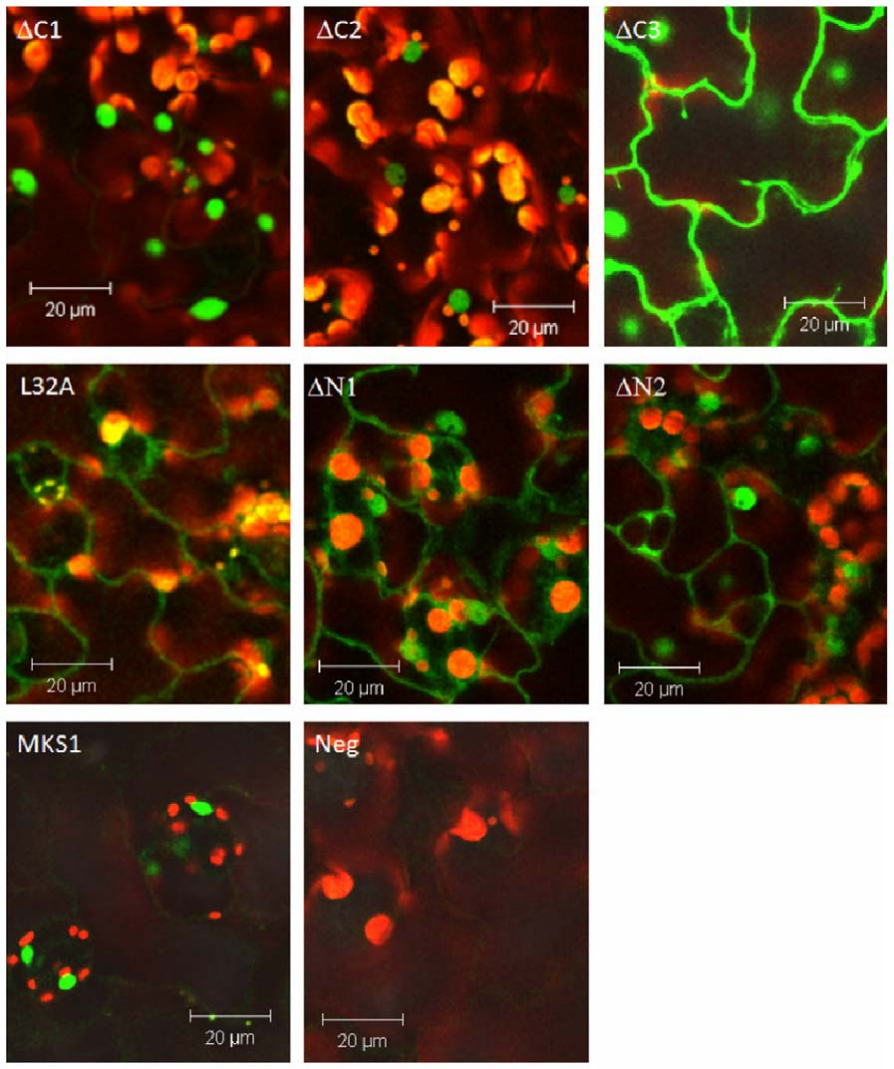
*in vivo* localization of MKS1 in leaf epidermal cells. Top row, left to right: MKS1ΔC1-GFP, MKS1ΔC2-GFP and MKS1ΔC3-GFP. Middle row, left to right: MKS1-L32A-GFP, MKS1ΔN1-GFP and MKS1ΔN2-GFP. Bottom row, left and right: MKS1-GFP and untransformed negative control.

MKS1 is exclusive localized in nuclei and MPK4 primarily localizes to the nucleus, consistent with their ability to interact *in vivo* [7]. To examine the subcellular localization of the different mutant versions of *MKS1*, we analyzed the localization of the MKS1-L32A point mutation and the five different truncations of MKS1 by gene fusions to green fluorescent protein (GFP). To confirm that intact GFP-MKS1 fusion proteins were produced, immuno-blots were performed using anti-GFP antibody (Figure S2). Full length MKS1 produces strong GFP fluorescence in the nucleus (Figure 6, MKS1). The C-terminal truncations MKS1ΔC1 and MKS1ΔC2 also localized almost exclusively in nuclei (Figure 6, ΔC1 and ΔC2), whereas MKS1ΔC3 and the two N-terminal truncations, MKS1ΔN1 and MKS1ΔN2, displayed strong fluorescence in both nuclei and cytoplasm (Figure 6, ΔC3, ΔN1 and ΔN2). Interestingly, MKS1-L32A only seemed to display weak fluorescence in the nucleus and was primarily localized to the cytosolic compartment (Figure 6, L32A). These results indicate that the region of MKS1 including residues 129-223 are dispensable for its specific nuclear localization. On the other hand, residues 1- 74 (MKS1ΔC3) alone do not produce specific nuclear localization. This indicates that the putative docking domain alone does not specify nuclear localization, but that the VQ domain is needed together with the docking domain for nuclear localization.

### An intact N-terminus is required for MKS1 function

**Figure 7:**
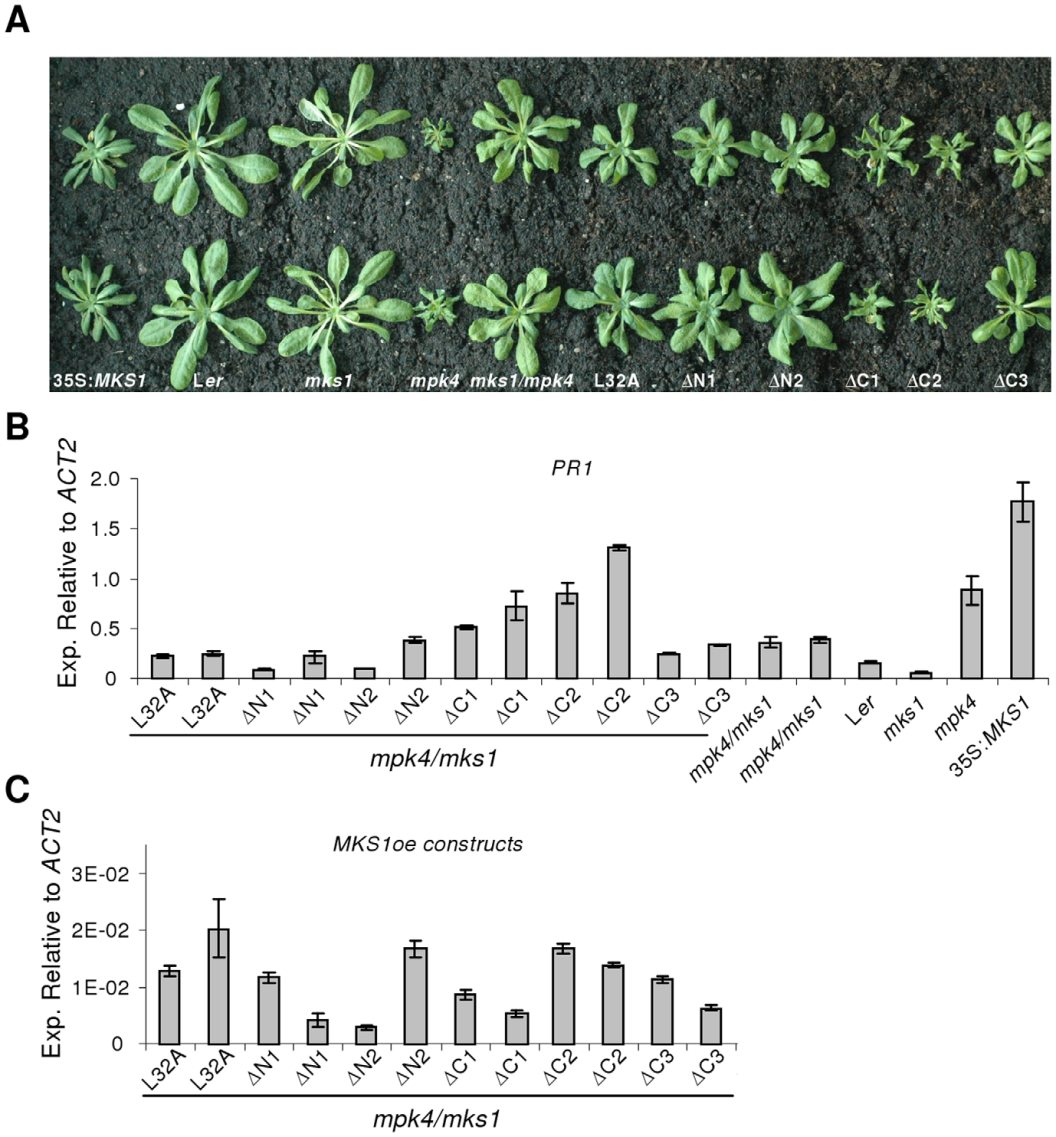
Effects of transgenic expression of MKS1 constructs in *mpk4*/*mks1*. (**A**) Phenotypes of three week-old plants with the indicated genotypes were grown under short-day conditions. MKS1-L32A, MKS1ΔN1, MKS1ΔN2, MKS1ΔC1, MKS1ΔC2 and MKS1ΔC3 are overexpressed with the constitutive 35S promoter in *mpk4*/*mks1* double mutants. Plants were grown on soil at 22°C and photographed 3 weeks after planting. (**B**) and (**C**) Real-time PCR detection of *PR1* (**B**) and *MKS1oe* (**C**) mRNA. Samples were tested in triplicate and normalized to *ACTIN2*. Means ± SD are shown.

To examine the biological significance of the MKS1-L32A point mutation and the five different truncations of MKS1, we transformed these six constructs into *mpk4*/*mks1*double mutant to assess their ability to complement *mks1*. As noted in Figure 1C, *mks1* partially suppressed the *mpk4* phenotype, and re-introducing functional MKS1 reverted *mpk4/mks1* to the *mpk4* dwarf phenotype. As can be seen in Figure 7A, only MKS1ΔC1 or MKS1ΔC2 truncations of MKS1 reverted the *mpk4*/*mks1* phenotype to the *mpk4* dwarf phenotype. These results indicate that a functional MKS1 protein requires an intact N-terminus important for both MPK4 and WRKY33 interactions. To confirm that intact proteins were produced in these experiments, immuno-blots were performed using anti-MKS1 (Figure S3). However, MKS1ΔN1 and MKS1ΔN2 are truncated within the N-terminal sequence against which the anti-MKS1 antibody was raised and are therefore not recognized. MKS1ΔC3 was also not recognized by the anti-MKS1 antibody, although this truncation includes the sequence used to raise the anti-MKS1 antibody. An explanation may be that MKS1ΔC3 is not folded correctly and therefore not recognized by the antibody, because MKS1ΔC3 expressed in *E. coli* was also not recognized although the mRNA was detected by RT-PCR (data not shown).

Because overexpression of *MKS1* leads to increased expression of *PR1* [7], we examined whether ectopic expression of the point mutation construct MKS1-L32A or the truncations of MKS1 would have any effect on *PR1* expression (Figure 7B). Only transgenic plants overexpressing the C-terminal truncations MKS1ΔC1 and MKS1ΔC2 exhibited high *PR1* expression, with levels comparable to those in *mpk4* or 35S:*MKS1*, whereas MKS1ΔC3, MKS1ΔN1, MKS1ΔN2 and MKS1-L32 all showed similar *PR1* expression levels as *mpk4*/*mks1* or L*er* wild-type (Figure 7B). To confirm that *PR1* expression does not correlate with transgenic *MKS1oe* levels, we next analyzed the expression levels of the different transgenic *MKS1* versions. This showed that *PR1* mRNA levels are independent of the mRNA levels produced by the transgenic *MKS1oe* constructs, indicating that the increased *PR1* mRNAs do not simply reflect the levels of the transgenic *MKS1oe* mRNAs (Figure 7C).

## Discussion

We show that a loss-of-function *mks1* insertion allele is a much stronger suppressor of *mpk4* than *MKS1*-RNAi. In addition, we found that this *mks1* mutant exhibits decreased basal resistance to biotrophic pathogens, an effect not seen in plants with reduced accumulation of *MKS1* mRNA via RNAi. These observations are important because they demonstrate that MKS1 plays a role in maintaining basal resistance at levels similar to that found for other resistance signaling proteins like EDS1 and PAD4 [18]. That MKS1 plays important roles in plant resistance responses is further supported by the recent finding that the *snc4-1D* mutant, which exhibits increased resistance responses, is partially suppressed by *mks1* [19].

MKS1 interacts with both MPK4 and WRKY33. Using directed yeast two-hybrid screens with different MKS1 deletion forms, we found that the N-terminal domain, which contains a putative kinase docking domain, interacts with MPK4, whereas WRKY33 was found to interact with a plant-specific VQ motif-containing domain. MKS1 is a member of a small protein family sharing this conserved VQ motif of unknown function, but other than this region their primary structures are highly diverse. Two other VQ motif-containing proteins have been characterized: SIB1 is a nuclear-encoded protein that is targeted to chloroplasts and interacts specifically with plastid RNA polymerase σ-factor Sig1 [20], and AtCaMBP25 is a calmodulin-binding protein involved in abiotic stress tolerance [21]. Interestingly, SIB1 was also found to be induced by SA [22], and to be involved in disease resistance [11]. Thus, the *sib1* loss-of-function mutation is compromised in the induction of some defense-related genes triggered by pathogen infection, whereas over-expression of *SIB1* activates defense-related gene expression following pathogen infection, leading to enhanced resistance to infection by *P. syringae*. It is thus tempting to speculate that like MKS1 and SIB1, other VQ domain-containing proteins also play roles in basal resistance. Future studies, including double mutant analysis and protein interaction experiments, will clarify the role this protein family may exert in plant immunity and whether MKS1 and SIB1 play similar roles in the MPK4 regulatory node.

We previously showed that MKS1 is localized in nuclei and interacts with MPK4 in this compartment [7], [8]. The in-depth analysis of MKS1 constructs presented here demonstrates that the N-terminal part of MKS1 is important for MPK4 interaction. In addition, a single amino acid change, L32A, within the putative MPK4 docking motif of MKS1, is sufficient to reduce MKS1 and MPK4 interaction and to exclude MKS1 from nuclei. In addition, expression of MKS1 L32A fails to revert the phenotype of the *mpk4/mks1* double mutant to that of the single *mpk4* mutant. Therefore, Leu32 of MKS1 appears to be essential for all known aspects of MKS1 function, indicating that the integrity of the MKS1 N-terminal is essential for proper function.

An intact N-terminus including a putative MAPK docking domain and a VQ motif seems to be required for MKS1 to revert *mks1*/*mpk4* to the *mpk4* dwarf phenotype as visualized by the phenotypic inspection of *mks1*/*mpk4* plants expressing MKS1-L32A, MKS1ΔN1, MKS1ΔN2 and MKS1ΔC3. In contrast, amino acids 127-223 comprising the C-terminus are dispensable for this effect. These results solidify the importance of the N-terminal region for MKS1 function. As noted above, domain 2 contains a VQ motif and is required for interaction with WRKY33, suggesting that WRKY33 is involved in the *mpk4* dwarf phenotype. As observed in the reversion effects for *mks1*/*mpk4*, correct MKS1 nuclear localization also depends on an intact docking domain, as evidenced by the increased cytoplasmic localization of MKS1-L32A, MKS1ΔN1, MKS1ΔN2 and MKS1ΔC3.

## Materials and Methods

### Plant Materials and Growth Conditions

*mpk4* [5], *mks1* (GT.108403; [8]) and the *mpk4*/*mks1* double mutant, all in Landsberg (L*er*) background, were grown in chambers under short-day (8 h light/16 h darkness). Day and night temperatures were 21 and 16°C, respectively.

### Genotyping

The following gene-specific primers were used for PCR-based genotyping for *mpk4*: MPK4fwd: 5′-CAGAGATGCTGATGATTAGTAACA-3′and MPK4rev: 5′- GGATCCATGTCGGCGGAGAGTTGTTTC-3′ and for *mks1*: MKS1fwd: 5′- ATCTGGCGGCGGTGGAGATGT-3′ and MKS1rev-3′UTR: 5′- GCTACTACATTGGATATGTCA-3′. DS1: 5′-GTTTTCGTTTCCGTCCCGCAAG-3′ recognizes the Ds transposon insertion in *mpk4* and *mks1*.For genotyping the MKS1 truncations and the MKS1-L32A point mutation, the following primers were used: MKS1fwd: 5′- ATCTGGCGGCGGTGGAGATGT-3′ and MKS1rev-3′NOS 5′- CGGCAACAGGATTCAATCTT-3′. For the truncation MKS1ΔC3, MKS1fwd-ΔC3: 5′-TCCTTCCGATCAACAGAACCAGAAG -3′ and MKS1rev-3′NOS 5′- CGGCAACAGGATTCAATCTT-3′were used.

### Bacterial Growth Assays

Five-week-old plants were infiltrated with *Pseudomonas syringae tomato* DC3000 and growth assays performed according to [23]. Infections with *Hyaloperonospora arabidopsidis* were performed using 10^5^ conidia/mL under conditions described previously [24].

### Plasmid Construction and *Arabidopsis* Transformation

For yeast two-hybrids, the five truncated versions of *MKS1*, *MPK4* and *WRKY33* cDNA were amplified by RT-PCR from wild-type L*er* using the primers listed in Table S1. Hereafter, the truncated versions of *MKS1* were cloned into the *Nco*I and *Eco*RI sites of pGBKT7 and *MPK4*, *WRKY25* and *WRKY33* cDNA were cloned into the *Nco*I and *Eco*RI sites of pGADT7.

For transgenic analyses, the five truncations of MKS1 and the MKS1-L32A point mutant were cloned into the *Not*I site of the binary vector pJL12 using primers listed in Table S2. The pJL12 vector was derived from the binary vector pCB302-3 [25]. pCB302-3 contains two *Not*I sites, one in MCS2 and another in the vector backbone. In pJL12, the *Not*I site in the vector backbone has been deleted by a silent mutation. The six different pJL12-MKS1 vectors were transformed into *Agrobacterium* and subsequently into *mpk4*/*mks1* doubly homozygous plants by floral dipping [26]. Plants carrying the transgene were identified with BASTA^©^ selection and PCR.

For GFP fusion constructs, the five different truncations of MKS1 were cloned into the *Xma*I and *Nco*I sites of pEGFP using primers listed in Table S3.

### Site-directed Mutagenesis

Full-length *MKS1* cDNA was in the pGBKT7 vector. Site directed mutagenesis PCR was performed using Pfu polymerase (Promega) according to the manufacturer's protocol (Stratagene).

### Yeast Two-Hybrid Assays

MKS1 truncated versions were in the bait vector (pGBKT7) and MPK4, WRKY25 and WRKY33 were in the prey vector (pGADT7). For the 18 alanine substituted constructs, MKS1 was in the prey vector (pGADT7) and MPK4 in the bait vector (pGBKT7). Transformation of yeast strain AH109 and yeast two-hybrid assays were performed as described by Clontech.

### Immune-blot and Antibodies

Soluble protein was extracted by grinding plant material in liquid N_2_. 600 µL buffer (100 mM Tris•Cl, 150 mM NaCl, 1 mM EDTA, 0.1% Triton X-100, 0.05% SDS, 10% glycerol, 5 mM DTT, 1x Complete Protease inhibitor) were added to 0.5 mL ground material and vortexed thoroughly. Insoluble material was removed by centrifugation at 500 g for 10 min. Protein concentration in the supernatant was measured with Bradford Reagent (Biorad). Protein was transferred from SDS-PAGE gels onto Hybond ECL nitrocellulose membranes (Amersham) using a semi-dry blotter (BioRad) and standard reagents. Membranes were blocked using 5% skimmed milk powder, 0.04% Tween-20 in PBS. Mouse monoclonal and polyclonal antibodies were against MKS1 (HYB 330-01; Statens Serum Institut, Denmark). HRP conjugated monoclonal goat anti-Mouse IgG was from DakoCytomation. HRP conjugated antibody was visualized using the Supersignal West Pico kit (Pierce).

### RNA Preparation and Quantitative RT-PCR

RNA was isolated from 3-week old plants grown under short day conditions using the RiboPure kit (Ambion) according to the manufacturer's instructions. Quantitative RT-PCR was performed as described previously [8].

### Confocal Microscopy

Analysis of GFP expression in mesophyll cells of young leaves was performed by confocal laser-scanning microscopy on a Zeiss LSM 510 confocal microscope equipped for epifluorescence analysis of GFP fluorescence.

## Supporting Information

Figure S1 MKS1 and homologs. Protein sequences of selected accessions were aligned at http://www.ebi.ac.uk/Tools/clustalw2/index.html and identical/similar residues highlighted at http://www.ch.embnet.org/software/BOX_form.html. The putative docking domain and VQ domain are indicated by overbars. At: Arabidopsis thaliana; Pt: Populus trichocarpa; Rc: Ricinus communis; Vv: Vitis vinifera; Zm: Zea mays; Sb: Sorghum bicolor; Os: Oryza sativa.
(0.37 MB TIF)

Figure S2 Immuno-blot of total cellular proteins from transgenic plants expressing MKS1-GFP fusion constructs immunodetected with anti-GFP antibody. Truncation MKS1 ΔN2 is so highly expressed that a small fraction of the correct fusion is degradated into smaller fragments.
(0.38 MB TIF)

Figure S3 Immuno-blot of total cellular proteins from transgenic mpk4/mks1 plants expressing MKS1 truncations immunodetected with anti-MKS1 antibody. Several lines for each construct were tested and only lines with the expected protein size were used for further analyses. Truncations MKS1ΔN1, MKS1ΔN2 and MKS1ΔC3 are not recognized by the anti-MKS1 antibody.
(1.08 MB TIF)

Table S1 List of primers used for yeast two-hybrid assays.
(0.22 MB TIF)

Table S2 List of primers used for complementation studies.
(0.23 MB TIF)

Table S3 List of primers used for GFP fusion constructs.
(0.22 MB TIF)
