# Supplementary figures and images for: Arabidopsis MKS1 Is Involved in Basal Immunity and Requires an Intact N-terminal Domain for Proper Function
Source: PLoS One. 2010 Dec 28;5(12):e14364. doi: 10.1371/journal.pone.0014364 (PMC3010986; doi:10.1371/journal.pone.0014364)

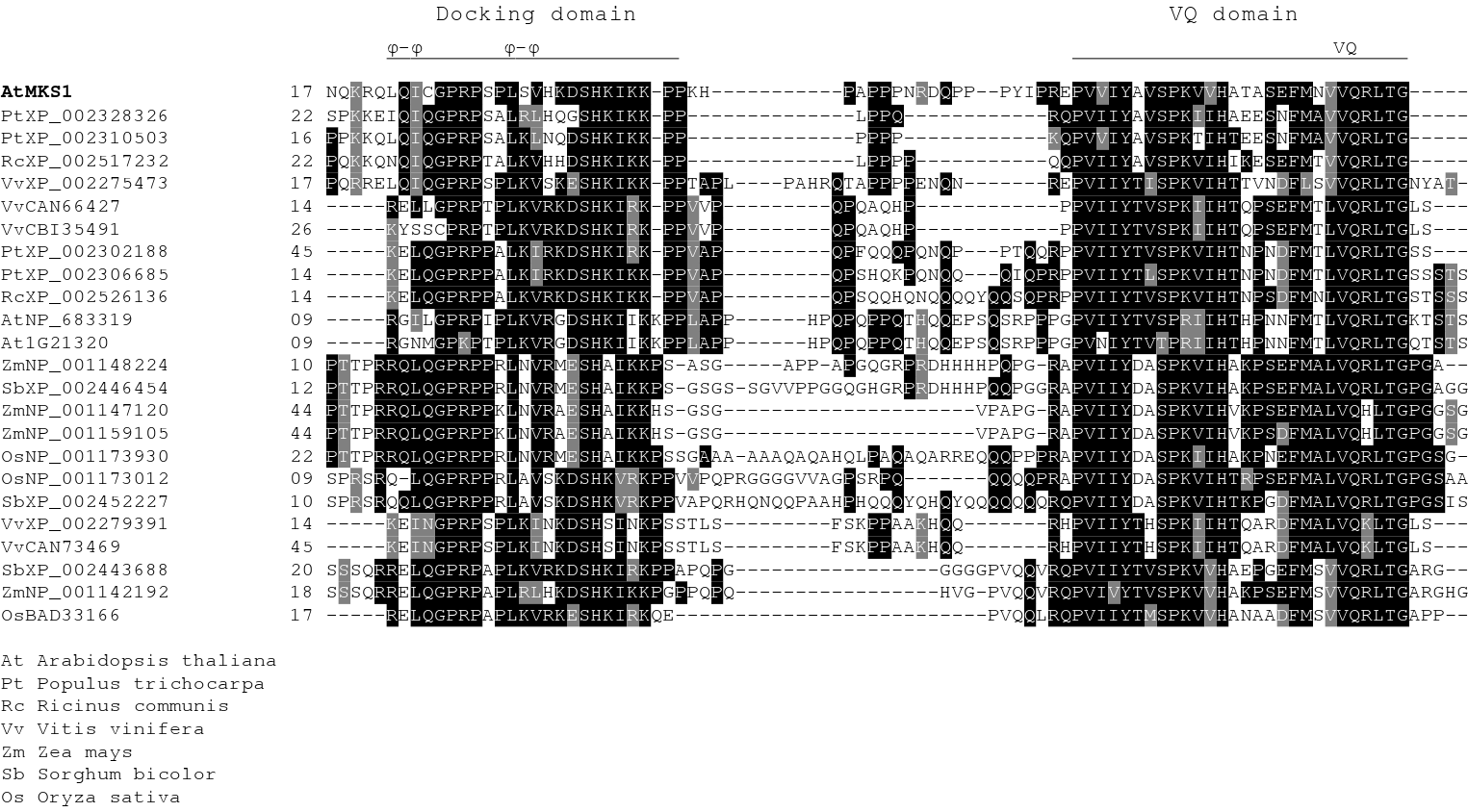

Supplement: Figure S1 — MKS1 and homologs. Protein sequences of selected accessions were aligned at http://www.ebi.ac.uk/Tools/clustalw2/index.html and identical/similar residues highlighted at http://www.ch.embnet.org/software/BOX_form.html. The putative docking domain and VQ domain are indicated by overbars. At: Arabidopsis thaliana; Pt: Populus trichocarpa; Rc: Ricinus communis; Vv: Vitis vinifera; Zm: Zea mays; Sb: Sorghum bicolor; Os: Oryza sativa. (0.37 MB TIF) [file pone.0014364.s001.tif]

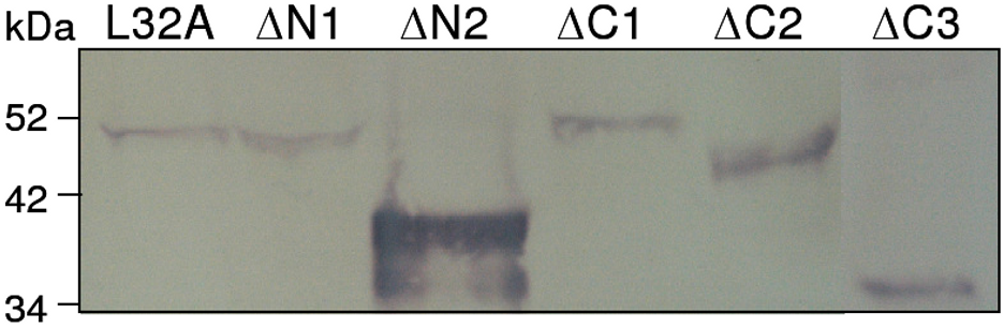

Supplement: Figure S2 — Immuno-blot of total cellular proteins from transgenic plants expressing MKS1-GFP fusion constructs immunodetected with anti-GFP antibody. Truncation MKS1 ΔN2 is so highly expressed that a small fraction of the correct fusion is degradated into smaller fragments. (0.38 MB TIF) [file pone.0014364.s002.tif]

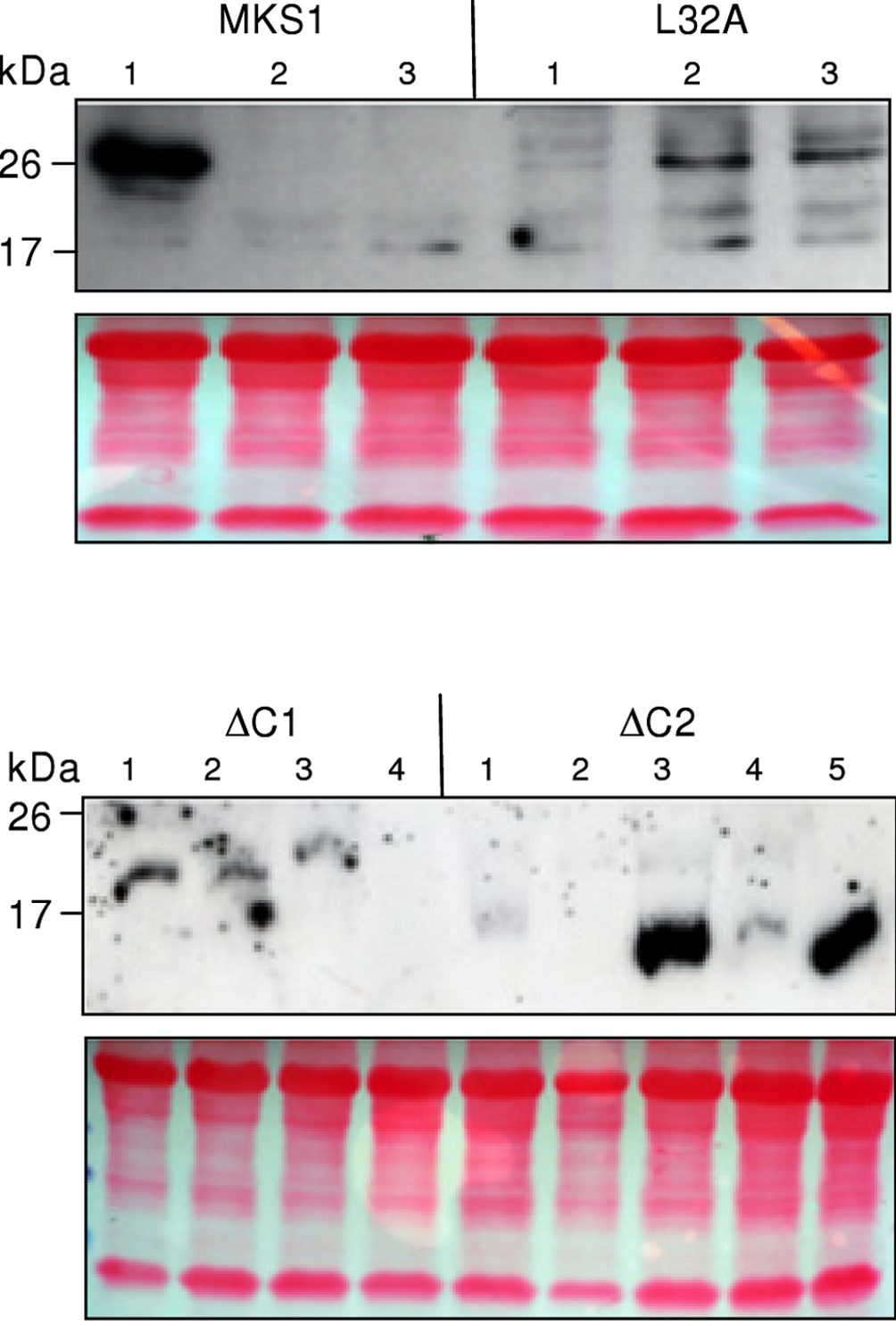

Supplement: Figure S3 — Immuno-blot of total cellular proteins from transgenic mpk4/mks1 plants expressing MKS1 truncations immunodetected with anti-MKS1 antibody. Several lines for each construct were tested and only lines with the expected protein size were used for further analyses. Truncations MKS1ΔN1, MKS1ΔN2 and MKS1ΔC3 are not recognized by the anti-MKS1 antibody. (1.08 MB TIF) [file pone.0014364.s003.tif]

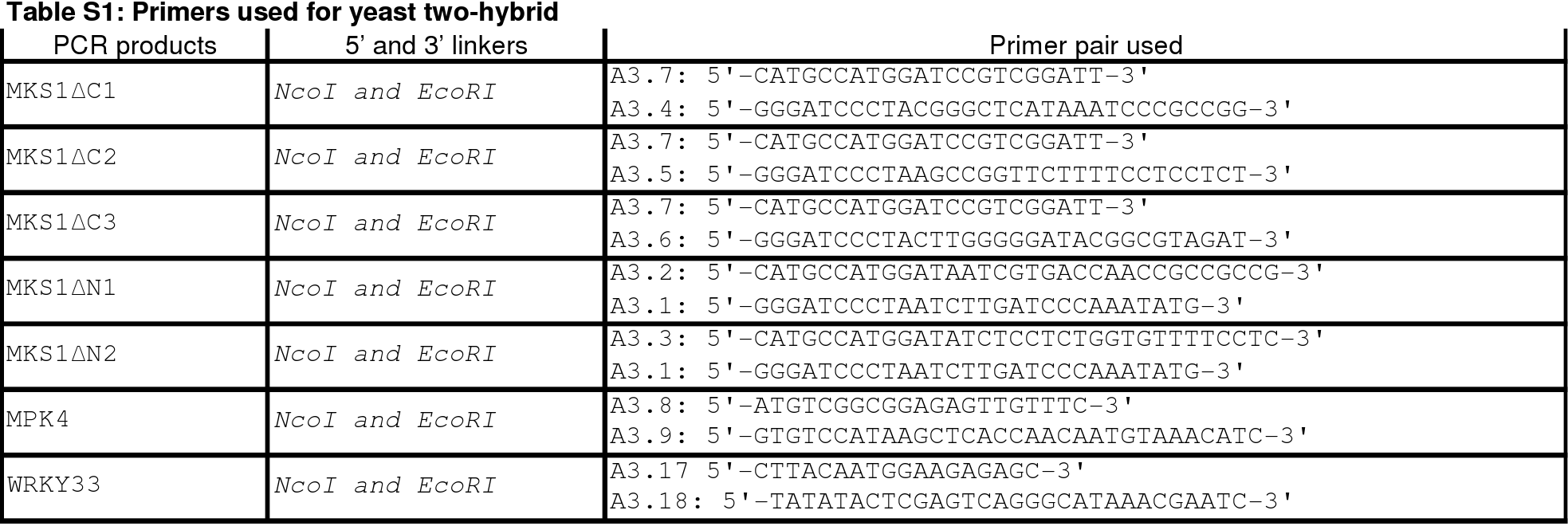

Supplement: Table S1 — List of primers used for yeast two-hybrid assays. (0.22 MB TIF) [file pone.0014364.s004.tif]

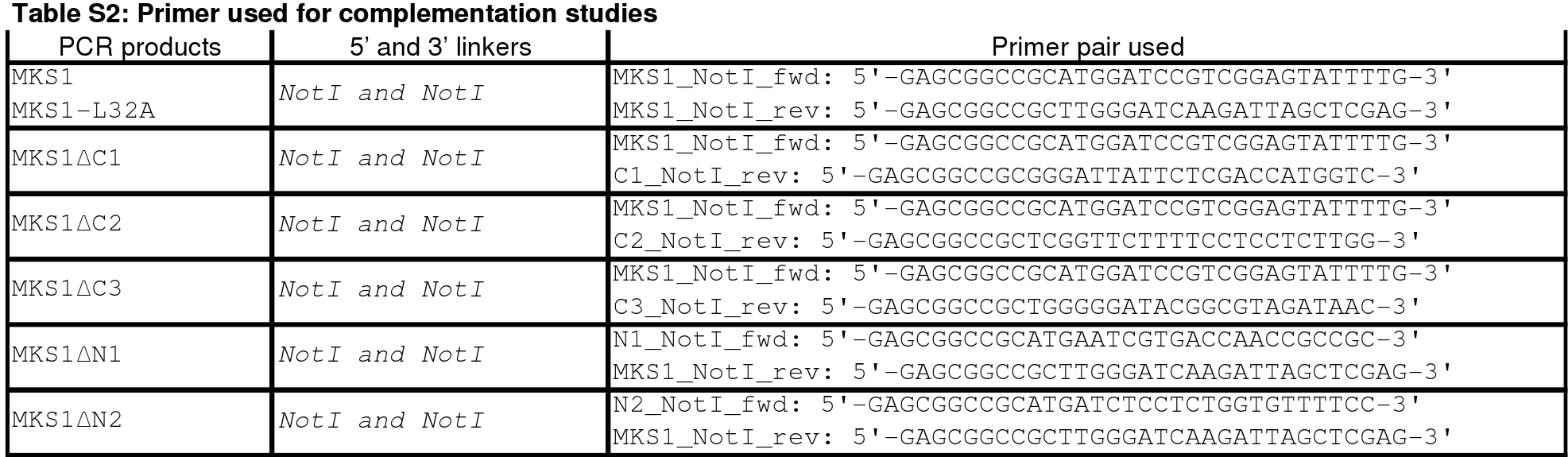

Supplement: Table S2 — List of primers used for complementation studies. (0.23 MB TIF) [file pone.0014364.s005.tif]

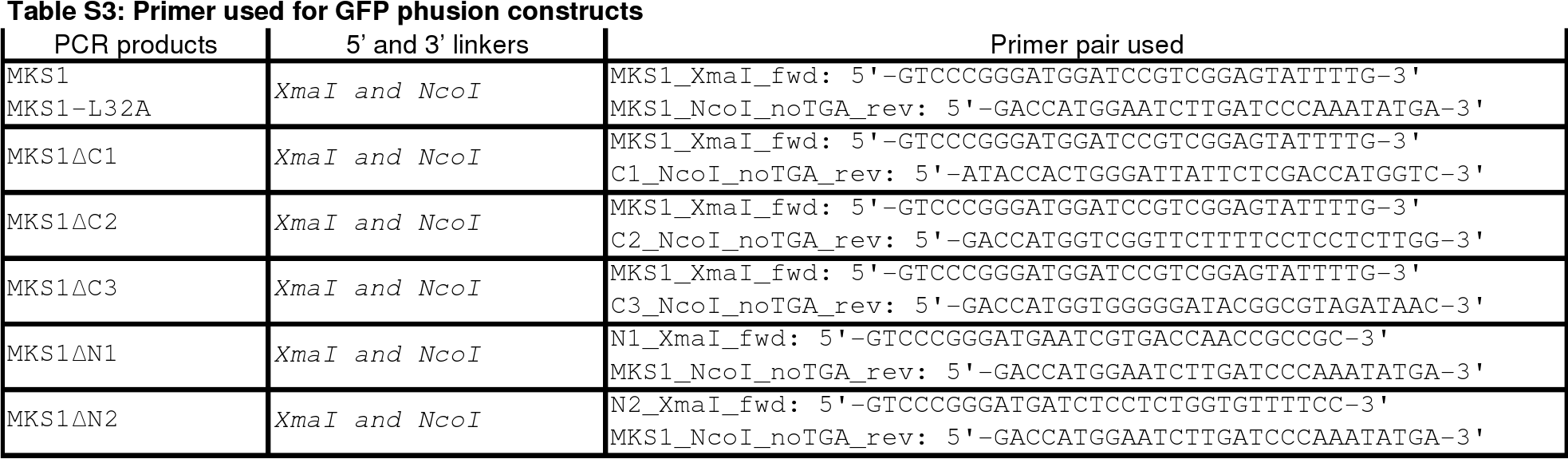

Supplement: Table S3 — List of primers used for GFP fusion constructs. (0.22 MB TIF) [file pone.0014364.s006.tif]
